# Laser Microdissection of Narrow Sheath Mutant Maize Uncovers Novel Gene Expression in the Shoot Apical Meristem

**DOI:** 10.1371/journal.pgen.0030101

**Published:** 2007-06-15

**Authors:** Xiaolan Zhang, Shahinez Madi, Lisa Borsuk, Dan Nettleton, Robert J Elshire, Brent Buckner, Diane Janick-Buckner, Jon Beck, Marja Timmermans, Patrick S Schnable, Michael J Scanlon

**Affiliations:** 1 Plant Biology Department, University of Georgia, Athens, Georgia, United States of America; 2 Cold Spring Harbor Laboratory, Cold Spring Harbor, New York, United States of America; 3 Center for Plant Genomics, Iowa State University, Ames, Iowa, United States of America; 4 Department of Statistics, Iowa State University, Ames, Iowa, United States of America; 5 Department of Plant Biology, Cornell University, Ithaca, New York, United States of America; 6 Division of Science, Truman State University, Kirksville, Missouri, United States of America; 7 Division of Mathematics and Computer Science, Truman State University, Kirksville, Missouri, United States of America; Salk Institute for Biological Studies, United States of America

## Abstract

Microarrays enable comparative analyses of gene expression on a genomic scale, however these experiments frequently identify an abundance of differentially expressed genes such that it may be difficult to identify discrete functional networks that are hidden within large microarray datasets. Microarray analyses in which mutant organisms are compared to nonmutant siblings can be especially problematic when the gene of interest is expressed in relatively few cells. Here, we describe the use of laser microdissection microarray to perform transcriptional profiling of the maize shoot apical meristem (SAM), a ~100-μm pillar of organogenic cells that is required for leaf initiation. Microarray analyses compared differential gene expression within the SAM and incipient leaf primordium of nonmutant and narrow sheath mutant plants, which harbored mutations in the duplicate genes *narrow sheath1 (ns1)* and *narrow sheath2 (ns2).* Expressed in eight to ten cells within the SAM, *ns1* and *ns2* encode paralogous WUSCHEL1-like homeobox (WOX) transcription factors required for recruitment of leaf initials that give rise to a large lateral domain within maize leaves. The data illustrate the utility of laser microdissection-microarray analyses to identify a relatively small number of genes that are differentially expressed within the SAM. Moreover, these analyses reveal potentially conserved WOX gene functions and implicate specific hormonal and signaling pathways during early events in maize leaf development.

## Author Summary

Unlike animals, plants exhibit a prolonged period of organogenesis, generating new leaves throughout their life cycle. This ability to maintain an embryo-like state is dependent upon the activity of shoot meristems, whose dual functions are to supply an inner core of pluripotent cells that sustain the shoot meristem while simultaneously generating new leaves derived from cells at the meristem periphery. Deciphering the complex combinations of molecular signals that transform meristematic cells into leaf primordia is a central question in plant developmental biology. In this study, we used the power of focused laser light to microdissect shoot meristems from neighboring leaf and stem tissue in the maize plant. Once isolated, we compared patterns of gene expression in normal shoot meristems to those of genetically mutant shoot meristems that form abnormal, narrow leaves. Out of more than 21,000 maize genes analyzed, 66 genes were identified as misexpressed in the mutant shoot meristems. All but one of the differentially expressed genes are previously unstudied in maize, and the majority are predicted to function during cell division, growth, or developmental signaling. Many of these novel genes are expressed in specific domains of the shoot meristem, consistent with their predicted function during maize leaf initiation.

## Introduction

**Figure 1:**
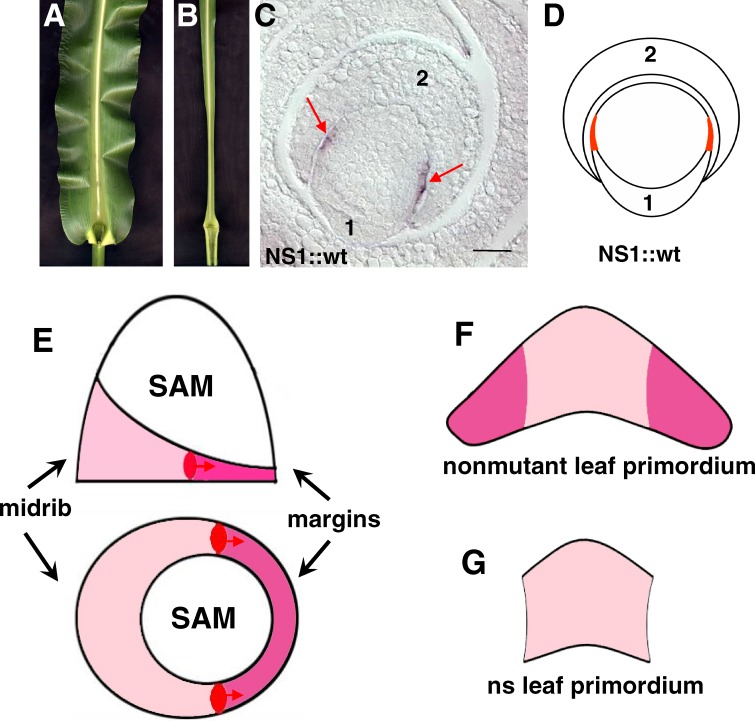
NS Mutants Delete Lateral Leaf Domains Due to Loss of NS Function in Lateral Foci of the SAM (A and B) The ns mutant leaf (B) contains a developmental deletion of a large lateral domain present in nonmutant leaves (A). In situ hybridization reveals NS1 transcript accumulation (C) and modeled in (D) in two lateral foci during initiation of a new leaf primordium (1) from the SAM. (E) Drawings of leaf initiation from the SAM are depicted in longitudinal (top) and transverse (bottom) sections. Leaf founder-cell initiation begins on one SAM flank that gives rise to the midrib and central domain of the leaf primordium (faint pink in [E–G]). NS1 function (red arrows in [E]) is required to complete founder cell recruitment of lateral domains of the leaf that includes the margin (dark pink in [E and F]). Loss of the NS1 recruitment function results in failure to recruit this lateral leaf domain (B) and (G). Images in (A, B, and C) are reproduced with permission from Nardmann et al. [2].

The paralogous *WUSCHEL1*-like homeobox (WOX) genes *narrow sheath1 (ns1)* and *narrow sheath2 (ns2)* function from two, lateral foci within the maize shoot apical meristem (SAM) (Figure 1) [1,2], which comprises a pool of over 1,200 pluripotent cells that ultimately generates all lateral organs of the vegetative shoot. Maize leaf development begins with the initialization of approximately 200 leaf founder cells in the SAM, a recruitment process whereby cells occupying the periphery of the SAM are signaled to become founder cells of the incipient leaf [3,4]. The *ns1* and *ns2* duplicate genes encode redundant functions during maize leaf development. Single mutations in either *ns* gene are nonphenotypic [1,2,5–7]. Plants harboring recessive mutations in both of the *ns* genes fail to initialize founder cells within a specific, lateral domain of the SAM; failure to transduce this founder-cell recruitment signal results in the preprimordial deletion of an extensive lateral domain from the mutant maize leaf (Figure 1) [6,7]. Although redundantly expressed in two foci comprising only approximately eight to ten total cells [2], NS1 and NS2 propagate a founder-cell recruitment signal throughout the lateral domain of the SAM from where much of the lower portion of the maize leaf is derived. The molecular nature of this recruitment signal and the mechanism of its transduction within the SAM are unknown.

The technique of laser microdissection (LM) permits the facile isolation of specific cells and tissues from plants [8]. Nanogram quantities of total RNA extracted from laser-microdissected tissues are linearly amplified by T7 RNA polymerase and used in microarray analyses. The combined use of LM and microarray technologies enables comparative analyses of discrete developmental fields while eliminating the transcriptional noise contributed by multiple tissues and downstream developmental events [9]. LM has been applied to global expression analyses of maize vascular and epidermal tissues, maize roots, as well as *Arabidopsis* embryos and floral organs [10–14]. The relatively large size of the maize SAM, approximately 200 founder cells are recruited into the incipient maize leaf versus 25–30 in *Arabidopsis* [3,15], renders the maize plant especially tractable to LM strategies owing to the unique utility of this new technology to microdissect localized gene expression patterns within plant tissues. Here we describe analyses of differential gene expression in whole maize SAMs derived from ns mutants and nonmutant siblings, genetically nearly identical tissues, whose differences stem solely from the loss of NS1 homeobox gene expression in approximately eight to ten cells in the lateral SAM domain.

In microarray analyses of more than 37,000 cDNAs representing approximately 21,721 maize genes, 66 genes are identified as differentially expressed in the ns mutant SAM, which demonstrates the power of LM-microarray to focus global analyses of gene expression to discrete, developmental domains. Quantitative real-time-PCR (qRT-PCR) corroborated the differential expression of 18 implicated genes and identified transcripts that are enriched in maize shoot meristematic tissues. In situ hybridization analyses revealed previously undescribed expression patterns for ten genes, six of which exhibit differential expression within the NS lateral domain of the SAM. Genes predicted to be involved in hormonal transport and signaling, signal transduction, and growth are especially implicated during NS-mediated leaf induction, and potentially conserved *WOX* gene functions during the regulation of two-component response pathways and of jasmonate-induced gene expression are identified.

## Results

### In the ns Mutant SAM, 66 Genes Are Differentially Expressed

**Figure 2:**
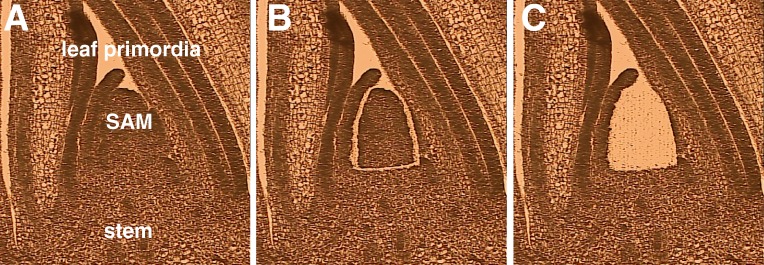
LM of the SAM from Paraffin Sections of Maize Seedlings (A) The apex before LM is shown. (B) Laser ablation is used to isolate the SAM from surrounding leaf primordia and stem tissue, without heating or damaging adjacent SAM tissues. (C) SAM tissue is microdissected via laser pressure catapulting, in which the laser is focused beneath the targeted SAM tissue and a high photonic force catapults the tissue into a collection tube suspended above the sample.

Whole SAMs, comprised of the meristem proper as well as the founder cells of the incipient leaf, were laser microdissected (Figure 2) from serial sections of ns mutant (genotype *ns1-R* and *ns2-R*) and nonmutant (genotype *Ns1/ns1-R* and *ns2-R*) seedlings grown under controlled conditions (see Materials and Methods). Although previous analyses revealed that NS1 and NS2 perform redundant functions during maize leaf development [1,2,5–7], the microarray experiments described herein measured differential gene expression conferred by NS1 function, given that the ns mutant and nonmutant samples analyzed in this study were all homozygous for the ns2-R mutation.

Technical barriers (described in Materials and Methods) precluded the use of our laser-microdissected RNA together with maize oligonucleotide microarrays. Therefore, following extraction and linear amplification of SAM RNA [11], expression profiles were generated for a combined total of 37,662 maize cDNA sequences (including approximately 21,721 maize genes) spotted onto three different microarray chips (SAM 1.1, SAM 2.0, and SAM 3.0), which were generated specifically for use in microarray analyses of the maize SAM (see Materials and Methods for descriptions of SAM chip contents; further details are provided at http://www.plantgenomics.iastate.edu/maizechip). SAM 3.0 in particular contains 10,816 sequences obtained from maize shoot apices (SAM plus four leaf primordia) as part of a SAM expressed sequence tag (EST) discovery program performed during this project [16].

We performed six biological replicates, each comprised of ten laser-microdissected SAMs from mutant and nonmutant seedlings. For each array platform, three of the six pairs were measured with Cy3 from mutant SAMs and Cy5 dye for nonmutant. Dye assignments were reversed for the other three replications. Cy5 minus Cy3 differences were computed for each slide following normalization. The differences were used to test for evidence of differential expression between mutant and nonmutant SAMs using a linear model analysis for each gene (see Materials and Methods) [17]. With the intent of focusing on genes most likely to be differentially expressed, 56 genes with *p*-values <0.001were selected; ten additional genes were selected with *p-*values between 0.001 and 0.01 and fold changes greater than 1.5 or less than 0.67 (Table 1).

**Table 1:**
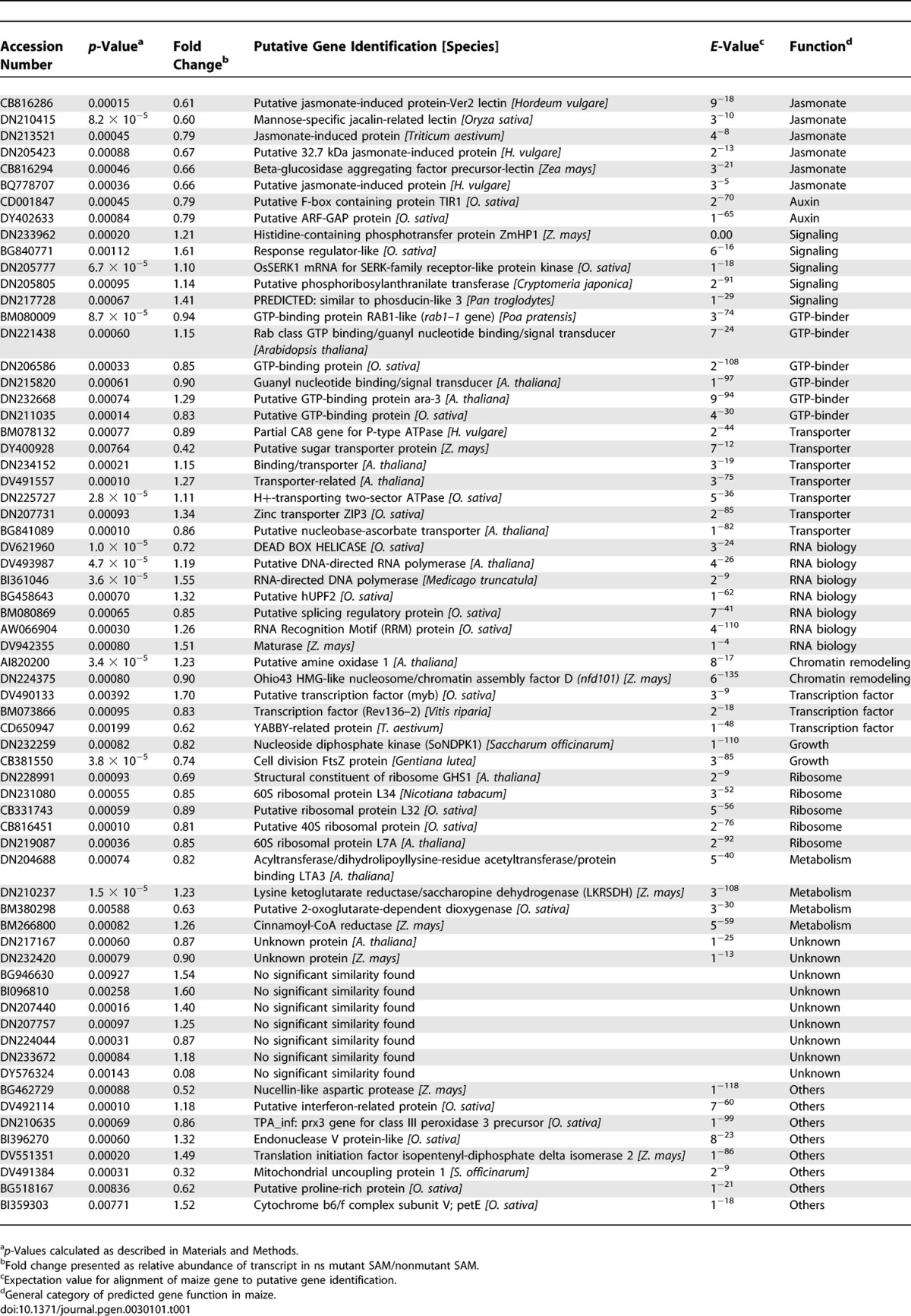
Genes Differentially Expressed in the ns1 SAM

**Figure 3:**
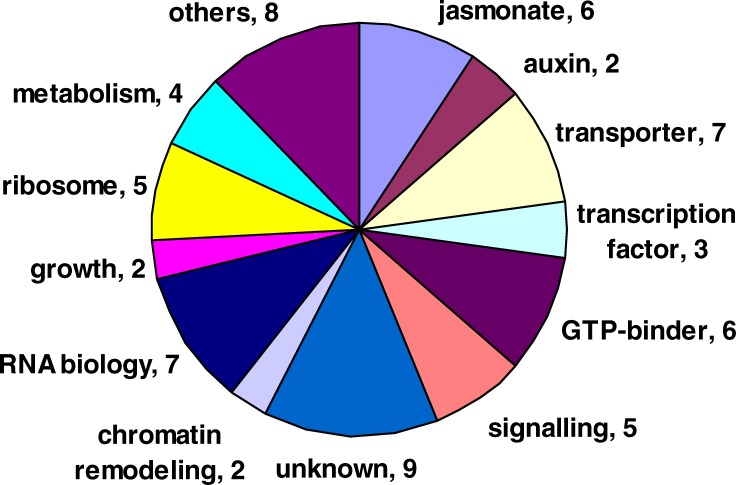
Predicted Functions of 66 Genes Differentially Expressed in the ns1-R Mutant SAM Excluding unknowns and housekeeping genes, 28/40 genes are predicted to function during hormonal-cellular signaling or growth and cell division.

**Figure 4:**
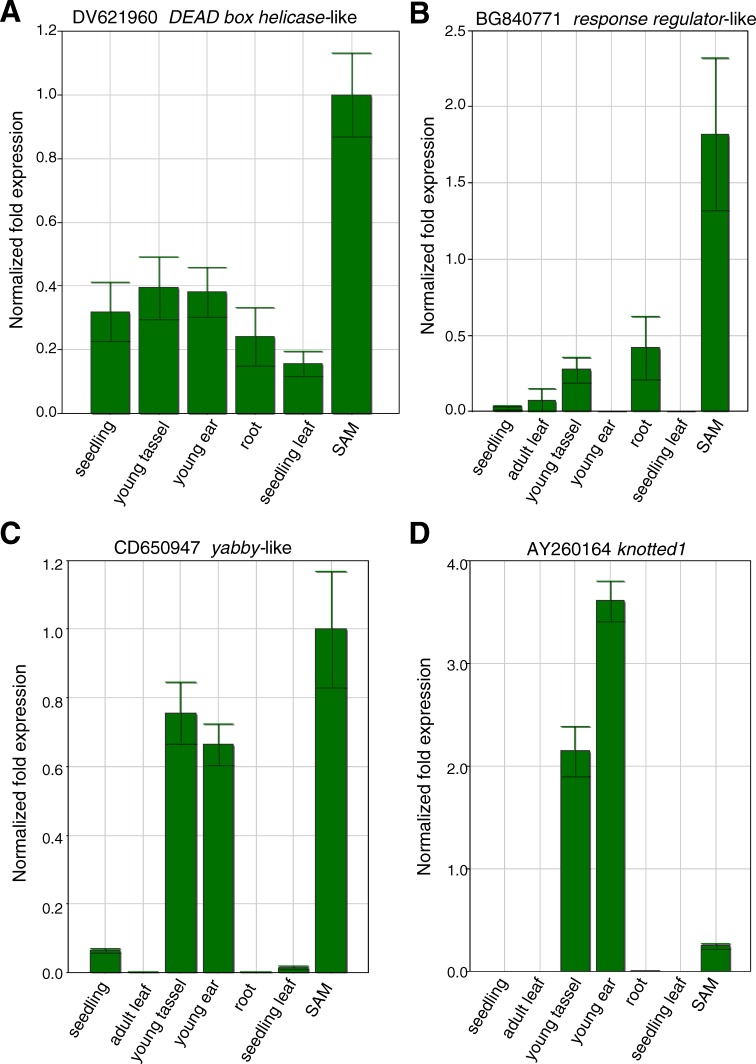
Identification of Shoot Meristem-Enriched Maize Transcripts by qRT-PCR Analyses of Multiple Maize Tissues (A) Transcripts of the *DEAD box helicase*-like gene DV621960 and the (B) *response regulator*-like gene BG840771 are enriched in the SAM, whereas (C) the *yabby-*like gene CD650947 is up-regulated in inflorescence tissues bearing floral meristems as well as in the SAM. (D) Control expression pattern of *knotted1* is shown*,* which is enriched in shoot meristems.

**Figure 5:**
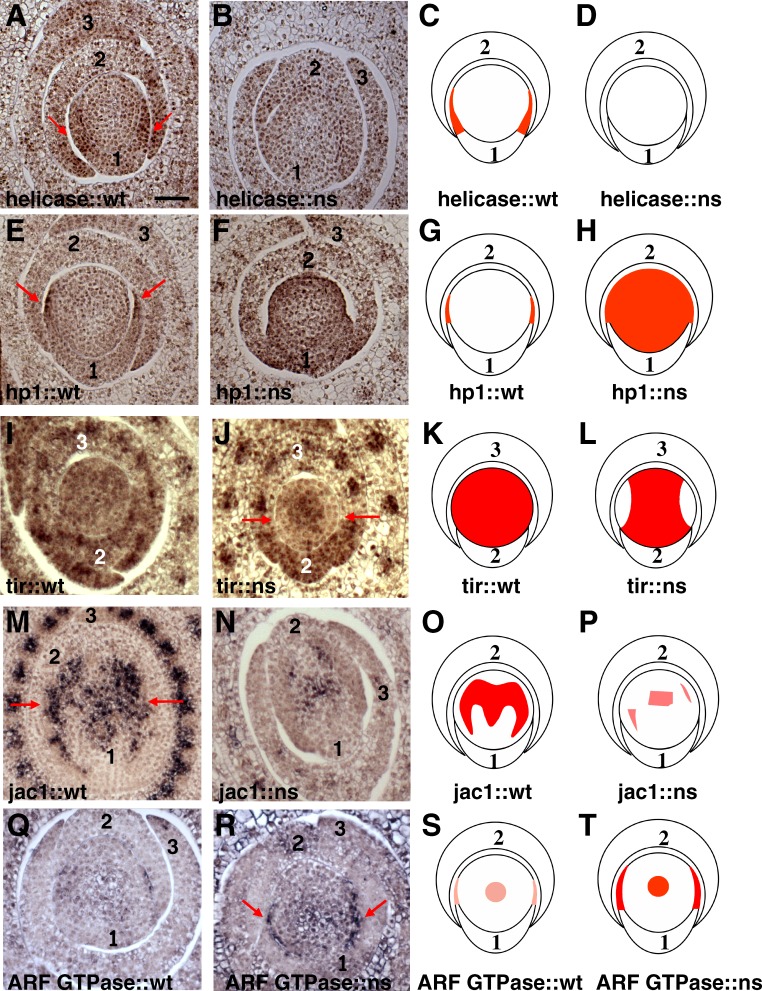
In Situ Hybridization Reveals Domain-Specific Expression of Differentially Expressed Maize Genes in Nonmutant and ns1-R Mutant Shoot Apices, Part I. Drawings of SAM expression patterns are modeled in (C and D), (G and H), (K and L), (O and P), and (S and T). Differentially expressed maize genes shown in nonmutant (wild type: [A], [E], [I], [M], and [Q]) and ns1-R mutant (ns: [B], [F], [J], [N], and [R]) shoot apices. No expression in leaf primordia is portrayed in cartoons, since transcripts accumulating in leaves were not LM sampled or reflected in microarray data. Probes were made from maize ESTs: DV621960 (A and B), CD001847 (E and F), DN210415 (I and J), *Zmhp1* (M and N), and DN221438 (Q and R). Predicted functions are abbreviated as: helicase, DEAD BOX HELICASE; hp1, HISTIDINE PHOSPHOTRANSFER PROTEIN1; tir1, TRANSPORT INHIBITOR RESPONSE F-BOX protein; jac1, JACALIN-related LECTIN; and GTPase, Rab class GTP-BINDING SIGNAL TRANSDUCER. Numbers denote leaf primordia. Analyses of expression patterns are provided in the text.

**Figure 6:**
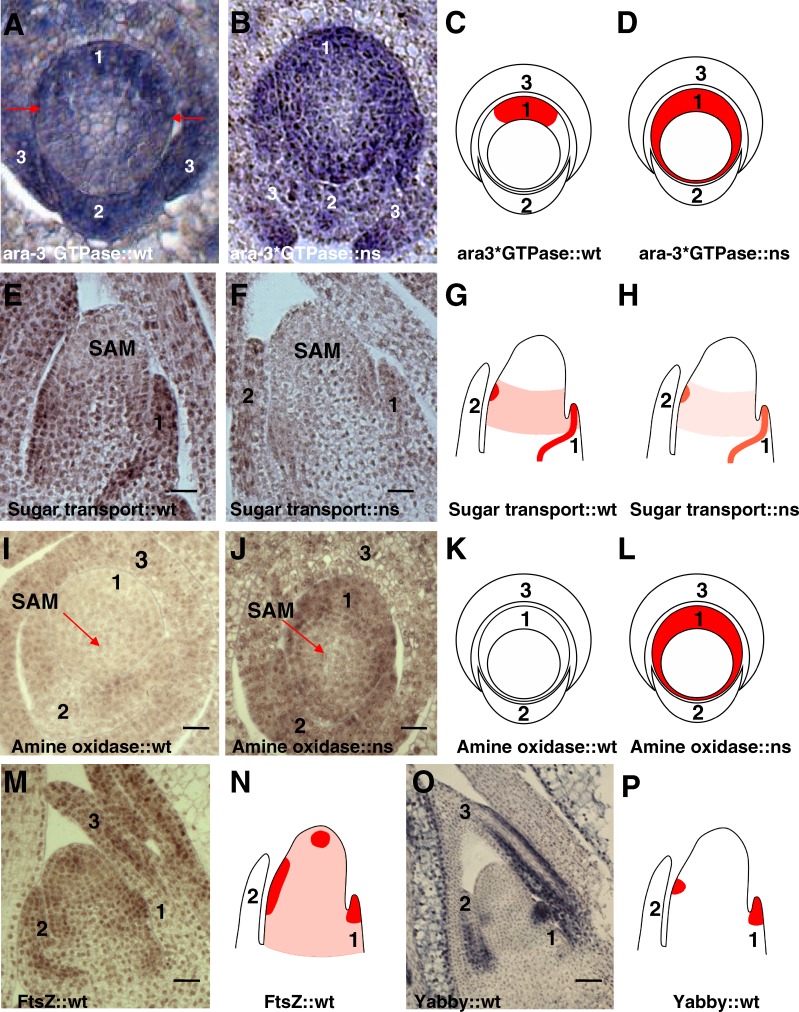
In Situ Hybridization Reveals Domain-Specific Expression of Differentially Expressed Maize Genes in Nonmutant and ns1-R Mutant Shoot Apices, Part II Drawings of SAM expression patterns are modeled in (C and D), (G and H), (K and L), and (N and P). Differentially expressed maize genes are shown in nonmutant (wild type: [A], [E], [I], [M], and [O]) and ns1-R mutant (ns: [B], [F], and [J]) shoot apices. No expression in leaf primordia is portrayed in drawings, since transcripts accumulating in leaves were not LM sampled or reflected in microarray data. Probes were made from maize ESTs: DN232668 (A and B), DY400928 (E and F), AI820200 (I and J), CB381550 (M), and CD650947 (O). Predicted functions are abbreviated as: ara GTPase, GTP-BINDING PROTEIN ARA-3; sugar trans, SUGAR TRANSPORTER PROTEIN; amine oxidase, AMINE OXIDASE1; FtsZ, CELL DIVISION FTSZ PROTEIN; and Yabby, YABBY-RELATED PROTEIN. Numbers denote leaf primordia. Analyses of expression patterns are provided in the text.

Bioinformatic predictions of function were performed for all genes differentially expressed in ns mutant apices and are presented at Gene Expression and Visualization Application (GENEVA, http://sam.truman.edu/geneva/geneva.cgi), a SAM gene-expression database created during this project [18]. A total of 11 functional categories are identified (Figure 3), including genes predicted to be involved in two-component signaling, auxin transport and signaling, jasmonate-induction/sugar signaling, intercellular transport, RNA processing, chromatin remodeling, transcription regulation, growth/cell division, ribosome structure, and general metabolism. Of the 66 genes identified in our microarray analyses, just one *(Zmhp1)* has been characterized biochemically [19], and none have been subjected to genetic analysis in maize. In addition, nine genes of unknown function are differentially expressed in ns mutant SAMs (Table 1). Excluding unknowns and genes predicted to be involved in general metabolism or “housekeeping” function, 28 out of the remaining 40 genes are predicted to be involved in some aspect of signal transduction or cell differentiation/growth. To ensure that the cells comprising the SAM lateral domains were not injured or degraded due to proximity to the ultraviolet laser during microdissection, internal controls included comparisons of *ns2* transcript abundance in laser-microdissected SAMs versus whole seedlings. qRT-PCR and microarray data confirmed that *ns2* expression is indeed enriched in the laser-microdissected SAM samples (unpublished data), suggesting that the SAM lateral domains are not compromised by the ultraviolet laser-microdissection procedure. The identification of additional, previously uncharacterized gene expression within the SAM lateral domain (Figures 4A, 5, and 6; as described below) provides further proof that these SAM domains were not destroyed during microdissection.

### qRT-PCR and In Situ Hybridizations Are Used to Corroborate Microarray Data and Identify SAM Domain-Specific Differential Expression

qRT-PCR of cDNA prepared from RNA microdissected from ns and nonmutant SAMs corroborated the differential expression of 18 out of 22 genes tested, whose microarray fold changes were large enough to detect by qRT-PCR methodology (i.e., fold changes ≤0.67 and ≥1.5) (Table 2). Overall, the qRT-PCR and microarray data exhibited remarkable agreement in quantitative fold change between ns mutant and nonmutant SAMs (Pearson correlation coefficient was 0.856, *r* ^2^ = 0.733, *p* = 0.000006). A total of four genes originally detected as differentially expressed in ns apices by microarray analyses could not be verified by qRT-PCR and were removed from further consideration. One such false positive gene encoding a predicted F-Box protein (BM078718) may have been detected by cross-hybridization to a TIR1-like F-box gene (CD001847) that was later verified as differentially expressed via in situ hybridization (described below) (Figure 5I–5L). This example illustrates one drawback to the use of long cDNA arrays in expression profiling and underscores the importance of secondary verification of microarray data by qRT-PCR and in situ hybridization.

**Table 2:**
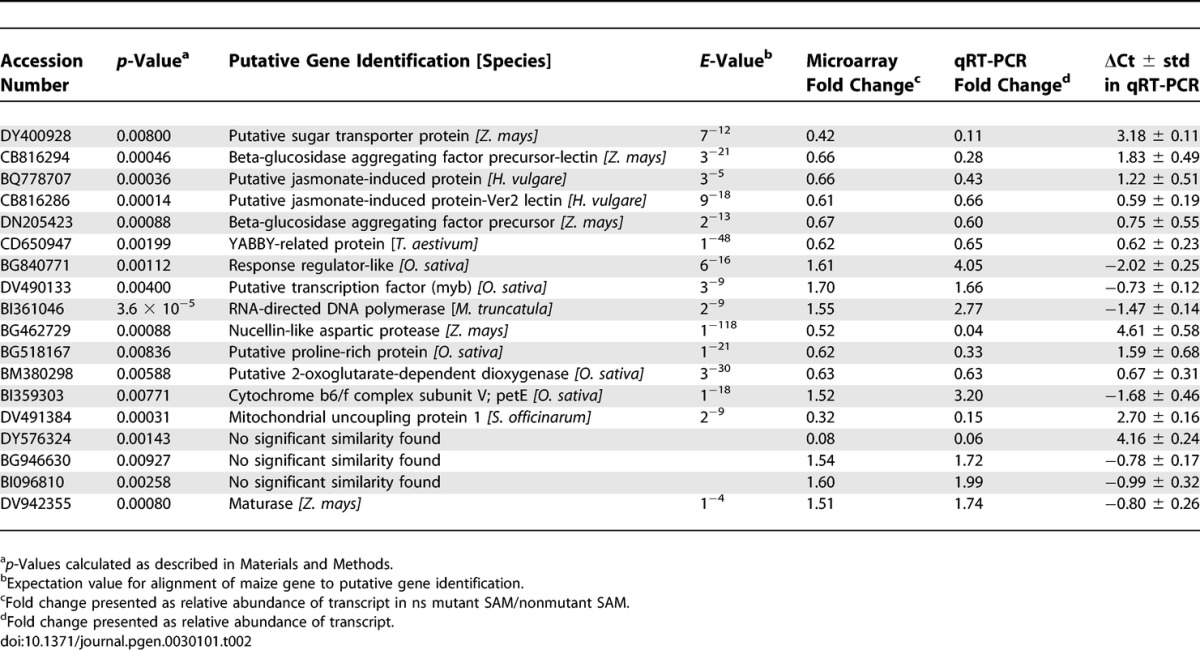
qRT-PCR Corroboration of ns1-R Differentially Expressed Genes

As part of a larger effort (http://maize-meristems.plantgenomics.iastate.edu) to identify maize genes whose expression is enriched in shoot meristems, qRT-PCR analyses of transcript accumulation in a variety of maize tissues were performed for thirteen genes contained in Table 1 (Figure 4; unpublished data). Maize tissues examined in these analyses included the vegetative SAM, immature tassel inflorescence, immature ear inflorescence, whole seedlings, expanded mature leaves, expanded seedling leaves, and seedling root (see Materials and Methods). In addition, in situ hybridization was used to characterize the expression patterns of 14 differentially expressed maize genes, four of which exhibited weak or indiscernible staining and could not be interpreted. A total of ten genes examined via in situ hybridization of maize apices yielded interesting mRNA accumulation patterns (Figures 5 and 6); six of which exhibited lateral SAM domain-specific differential expression and are thereby predicted candidate genes functioning within or nearby the NS expression foci (Figure 1C) during maize leaf initiation.

For example, a predicted *DEAD box helicase* gene (DV621960) is expressed in the lateral founder cell regions of the initiating nonmutant leaf primordium (red arrows in Figure 5A), whereas no expression is noted in ns mutant sibling SAMs (Figure 5A–5D). These in situ hybridization data are in agreement with our microarray data (Table 1), which indicated that expression of DV621960 is significantly down-regulated in ns apices (*p* = 1 × 10^−5^). Characterized by their shared ability to unwind RNA helices, the 32 DEAD box HELICASE proteins of *Arabidopsis* are implicated in a variety of RNA metabolic processes (including transcription and pre-mRNA splicing, ribosome biogenesis and translation, gene expression, and meristematic cell division), although their functions as yet described are specific and noninterchangeable [20,21]. Indeed, qRT-PCR analyses of DV621960 transcript accumulation in various maize tissues reveal that expression of this maize *DEAD box helicase-*like gene is enriched in the SAM (Figure 4A). Moreover, the lateral SAM domain-specific differential accumulation of DV621960 suggests that its expression is activated by NS function and is related to founder-cell recruitment. As described below, the lateral domain-specific differential expression of five additional maize genes (CD001847, DN210415, *Zmhp1,* DN221438, and DN232668) in the ns mutant SAM implies that domain-specific hormonal and signaling mechanisms are important during maize leaf initiation.

### Two-Component Signaling Pathway(s) Implicated in NS FUNCTION: SAM-Enriched and Domain-Specific Differential Expression

Two-component response regulators comprise an evolutionarily conserved signal transduction pathway involving the transfer of phosphate from a sensor HISTIDINE KINASE to a RESPONSE REGULATOR (ARR) effector molecule [22]. HISTIDINE PHOSPHOTRANSFER proteins mediate these exchanges, whereupon the phosphorylated ARR regulates the activation of specific cellular responses. In plants the two-component system is implicated in numerous developmental functions including responses to ethylene and cytokinin [23,24], whereas pseudo-response regulators function to control circadian rhythms [25]. Previously, microarray analyses of WUS1 induction revealed that four Arabidopsis arr genes are direct targets of WUS1 transcriptional repression [26].

Both the predicted maize response regulator gene BG840771 and the maize phosphotransfer gene *Zmhp1* are up-regulated in the ns mutant SAM (Table 1). qRT-PCR reveals that transcripts from the predicted response regulator gene BG840771 accumulate to 4.05-fold higher in the ns SAM (Table 2), which is consistent with our microarray data (Table 1). In addition, tissue-specific qRT-PCR revealed that transcripts of BG840771 are especially abundant in the vegetative SAM as compared to other maize tissues (Figure 4B). These data further illustrate the utility of LM-microarray analyses to identify new genes whose expression and implicated function are likely to be enriched in the SAM.

ZmHP1 has been shown to function in vitro as a phospho-donor for the maize response regulators (ZmRRs) ZmRR1, ZmRR4, ZmRR8, and ZmRR9, whereas accumulation of ZmHP1 in both the cytoplasm and nucleus is consistent with its predicted function during two-component signaling [19]. In situ hybridization of *Zmhp1* reveals transcript accumulation at two lateral foci within the nonmutant SAM (red arrows in Figure 5E and 5G), which mimics the *ns* expression domain (Figure 2C) [2]. In contrast, *Zmhp1* transcripts accumulate throughout the ns mutant SAM (Figure 5F and 5H); this up-regulated mutant expression is consistent with our microarray data (Table 1).

### Auxin Transport and Response Genes Are Differentially Expressed in the NS1-R SAM

Polar auxin transport is required for leaf initiation and lateral margin development in plants [27,28]. Auxin is transported to sites of leaf initiation via the PINFORMED1 (PIN) family of efflux proteins [29–32], a process that requires ADP-ribosylation factor (ARF)-GAP-mediated vesicular cycling of PIN proteins [33,34]. Auxin signaling involves targeted proteolysis of transcriptional regulators wherein the F-box protein TIR1 functions as an auxin receptor [35,36].

The two genes predicted to be involved in auxin biology are down-regulated in our analyses of ns mutant SAMs (Table 1), including a putative ARF-GAP encoding gene (DY402633) with predicted orthology to the *Arabidopsis van3/scf1* gene required for vesicle trafficking and PIN recycling [34,37] and the predicted maize orthologue of the *tir1* auxin receptor (CD001847). Whereas in situ hybridizations reveal that Zm*tir1 is expressed throughout the nonmutant SAM as well as in the margins and vasculature of leaf primordia (Figure 5I and 5K), Zm*tir1 transcript abundance is diminished specifically in the lateral domain of the ns mutant SAM (red arrows in Figure 5J and 5L). These data suggest that auxin transport and auxin signaling are correlated with NS-mediated recruitment of leaf founder cells within this lateral SAM domain.

### Jasmonate-Induced Sugar-Binding Genes Up-Regulated by NS Function

A total of six genes predicted to be induced by jasmonate, a phytohormone functioning during plant development and defense [38], are down-regulated in the ns mutant SAM (Table 1). Auxin signaling induces jasmonate responses and both hormones share common downstream signaling pathways [39–41], suggesting that down-regulation of jasmonate responses in ns apices might be related to defects in auxin signaling. Notably, all the jasmonate-induced genes identified herein encode putative lectins, carbohydrate-binding receptor proteins that are implicated during sugar transport in plants [42]. qRT-PCR corroborated the differential expression of four jasmonate-induced genes (Table 2), and in situ hybridization of a putative jacalin-related LECTIN encoding gene (DN210415) reveals a butterfly-shaped expression pattern at the insertion of the leaf primordium into the apex (Figure 5M and 5O). The “wings” of this expression pattern (red arrows in Figure 5M) are diminished or absent in ns mutants (Figure 5N and 5P), revealing down-regulated *lectin* mRNA accumulation in lateral domains of the mutant apex. Although transcript accumulation in leaf primordia was not measured in our microarray analyses, DN21415 transcripts are also detected in the vasculature of nonmutant leaves and are not detected in ns mutant leaf primordia. Notably, the apparent *Arabidopsis* orthologue of this particular jacalin-related *lectin* gene is known to be a direct target of the NS-related WOX protein WUS1 [26]. Furthermore, a putative sugar transporter-encoding gene (DY400928) was qRT-PCR-verified as down-regulated (0.11-fold compared to nonmutant) in the ns mutant SAM (Table 2), implying further that sugar transport and/or sugar signaling is required for NS function. In situ hybridization reveals accumulation of this putative *sugar-transporter* transcript in initiating leaf primordia and in leaf vascular traces within the nonmutant apex (Figure 6E–6H).

### Differential Expression of GTP-Binding Proteins Suggests That Multiple Signaling Networks Are Operating during NS-Mediated Recruitment of Leaf Founder Cells

Transcriptome analyses of ns mutant SAMs suggest that numerous GTP-binding proteins are involved during NS1 signaling within the initiating maize leaf. A total of six genes predicted to encode GTP-binding proteins are significantly misexpressed in the ns mutant (Table 1), including genes predicted to be involved in vesicle trafficking (three Rab GTPases and an ARF GTPase), signal transduction (a heterotrimeric G protein), and cell growth and division (a GTP1/OBG family GTPase) [43].

In the nonmutant SAM, transcripts encoding a putative Rab-class ARF-GTPase (DN221438) are detected in the meristem center, as well as in lateral stripes overlapping the NS functional domain (Figure 5Q and 5R). In the ns mutant SAM, the lateral expression of DN221438 is wider and more pronounced (red arrows in Figure 5R and 5T), consistent with the up-regulation observed in microarray hybridizations and implicating NS during negative regulation of this putative ARF-GTPase encoding gene. Likewise, a second predicted Rab-class GTPase encoding gene (DN232668) homologous to the *Arabidopsis* GTP-binding protein gene *ara3* [44] is also up-regulated in the ns mutant SAM (Table 1). In situ hybridization reveals that the *ara3-*like GTPase (DN232668) gene is expressed in the midrib/central founder-cell domain of the nonmutant SAM (red arrows in Figure 6A and 6C), whereas in mutant apices loss of NS function results in the expansion of this expression domain into the lateral SAM domain and thereby encompasses the entire founder-cell ribbon (Figure 6B and 6D).

### New Maize Genes Predicted to Be Involved in Chromatin Remodeling, Cell Division, and Growth Are Differentially Expressed in the ns Mutant SAM

We found two maize genes with predicted functions in chromatin remodeling (AI820200 and DN224375) that are significantly differentially expressed in ns mutant apices (Table 2). In situ hybridizations reveal that one such gene, encoding a predicted *amine oxidase* implicated in histone modification [45], is unexpectedly up-regulated in the midrib/central domain as well as in the lateral domain of ns mutant founder cells (Figure 6I–6L).

A maize *ftsZ*-related gene (CB381550) is significantly down-regulated in the ns mutant SAM (Table 1) and shares homology with *Arabidopsis* genes encoding tubulin-like, structural proteins required for cell division in chloroplasts [46]. In situ hybridizations of nonchlorophyllic maize apices reveal *ftsZ-*like transcript accumulation in actively dividing tissues including young leaf primordia and leaf founder cells, as well as in the SAM apical tip (Figure 6M and 6N). Although statistical parameters suggest robust support for differential expression of this gene in each of our six biological replicate samples (*p* = 3.8 × 10^−5^), no domain-specific differences in CB381550 expression are observed in situ hybridizations of ns samples. It is likely that in the absence of domain-specific changes in mRNA localization, our in situ hybridization protocols are unable to discriminate a 0.72-fold quantitative change in transcript accumulation.

Lastly, a previously undescribed maize *yabby-*like gene (CD650947), the putative orthologue of the *drooping leaf* gene of rice [47], is also down-regulated in our analyses of ns mutants. In nonmutant apices *yabby-*like transcripts accumulate in newly initiating leaf primordia (Figure 6O and 6P), an expression pattern that is consistent with the predicted role of YABBY proteins during initiation and expansion of lateral organ primordia [47–49]. No domain-specific changes in mRNA accumulation are noted in ns apices, however qRT-PCR corroborates the down-regulation of this maize *yabby*-like gene in the ns mutant SAM that was observed in microarray analyses (Table 2). Furthermore, tissue specific qRT-PCR reveals that transcripts of the *yabby*-like gene CD650947 accumulate in the SAM as well as in shoot meristem-enriched tissues such as the young tassel and young ear, each of which bears numerous spikelet and spikelet pair meristems (Figure 4C).

## Discussion

### LM-Microarray Is a Useful Technology to Identify Genes Expressed in Micro-Domains of Plant Tissues

The power of LM to focus microarray comparisons to small developmental fields is demonstrated in these analyses of ns mutant and nonmutant SAMs. Among the genes represented in this relatively small dataset are a number of likely candidates implicated during NS-mediated leaf initiation and predicted to function in hormonal signaling, signal transduction, or growth (discussed below). Although the primary developmental function of NS is shown to be localized in the shoot apex during recruitment of leaf founder cells in a lateral domain of the SAM [1,6,7], loss of NS function is predicted to cause widespread changes in gene expression in the growing seedling owing to enormous differences in the differentiation and expansion of marginal/lateral leaf tissues that happens during normal leaf development downstream of NS function (Figure 1A and 1B). The differential gene expression that ensues in mutant leaves following the ns-induced leaf domain deletion event is unlikely to address our experimental question, namely, the mechanisms of founder cell recruitment in the SAM.

Microarray analyses of the ns SAM provide a resource for new gene discovery; of the 66 differentially expressed genes identified in these analyses, all but one are previously undescribed maize genes, and at least three exhibit enriched transcription in maize shoot meristematic tissues (Figure 4). In addition, six candidate genes chosen for in situ hybridization analyses exhibited differential expression within or overlapping the SAM lateral domain of NS function and expression (Figures 1, 5, and 6) [1,2]. Our data illustrate the combined utility of in situ hybridization and LM technology to focus microarray comparisons to a discrete developmental field, thereby limiting the number of identified differentially expressed genes to those transcribed in close vicinity to the domain of NS function.

Although the use of six biological replicates enabled the selection of ns differentially expressed genes, it is noted that the majority of the candidate genes represented in Table 1 exhibit relatively modest fold changes in expression level. This result was expected, considering that the NS micro-domain of expression and function comprises a relatively small number of cells in the lower lateral region of the maize shoot apex (Figure 1C) [1,2]. During SAM LM (Figure 2) the RNA contribution of cells comprising the NS lateral domain is diluted by RNA collected from the rest of maize SAM, which may explain the relatively low fold changes observed in our microarray analyses. Unfortunately, without the use of technically prohibitive NS expression markers during LM of the SAM, reliable microdissection of the NS lateral micro-domain away from the rest of the SAM proper is not feasible.

### Analyses of NS Function Suggest That Multiple Hormonal and Signaling Pathways Are Involved in the Recruitment of Maize Leaf Founder Cells

Excluding genes of unpredicted function, the majority of transcripts differentially expressed in the ns SAM (28/40) are putatively involved in some aspect of developmental signaling or growth regulation (Table 1). These include genes involved in two-component response pathways, auxin signaling, jasmonate-induced pathways, as well as GTP-binding proteins implicated during signal transduction or cellular trafficking. These results are consistent with previous models for NS function during transduction of a cell-autonomous, founder-cell recruitment signal required for maize leaf initiation. Especially intriguing are those genes whose expression domains mirror or overlap the NS lateral foci (Figure 5; Figure 6A–6D). We hypothesize that these coexpressed genes may function closely downstream of NS. Subsequent reverse genetic and molecular/biochemical analyses of these implicated genes will test this hypothesis and help to elucidate the NS signaling pathway.

Our microarray data and in situ hybridization analyses of the maize *tir1*-like gene (CD001847; Figure 5I–5L) suggest that auxin activity is involved in NS-mediated recruitment of leaf founder cells in the lateral domain of the maize SAM. Previous analyses among multiple laboratories implicate PIN-mediated auxin transport during leaf initiation, and leaf initiation correlates with *knox* gene down-regulation within the SAM [50–52]. More recent studies established a mechanistic link between these two correlated phenomena, illustrating that *knox* down regulation requires auxin [28,53]. Initial analyses of NS function revealed that ns mutants fail to down-regulate KNOX accumulation in the lateral domain of the SAM, which correlates with the failure to recruit founder cells from this meristem domain [6,7]. In light of the recently established links between *knox* down-regulation, leaf initiation, and auxin activity, our microarray analyses and Zm*tir1 expression data suggest that NS-mediated founder-cell recruitment and KNOX down-regulation require TIR-mediated auxin signaling within the SAM lateral domain.

In addition, the expression of six genes predicted to encode jasmonate-induced lectins is consistently down-regulated in the ns mutant SAM (Table 1). Lectins are carbohydrate-binding proteins that function to facilitate the intercellular transport of sugars [42]. In situ hybridization of a jacalin-related *lectin* gene (DN210415) reveals a pattern of transcript accumulation that spreads laterally at the insertion site of the newly initiated leaf primordium into the shoot (Figure 5M–5P). The decreased lateral accumulation of DN210415 transcripts in ns mutant shoots suggests that NS promotes the novel expression pattern of this jasmonate-induced gene.

Likewise, a predicted sugar-transporter gene (DY400928) is under expressed in ns 1-R mutants (Figure 6E–6H; Tables 1 and 2), which further implicates a role for carbohydrate transport during NS function. Numerous studies reveal a hormone-like role for sugar signaling in plants, in which carbohydrate transport regulates gene transcription [54]. Subsequent analyses are required to determine if this putative sugar transporter and the jasmonate-induced lectins perform metabolic functions or signaling functions during NS-mediated founder-cell recruitment.

### Conserved *WOX* Gene Functions Suggested by Microarray Analyses of ns Mutant Apices

At present, *ns1* and its duplicated paralogue *ns2* are the only maize *wox* genes for which genetic analyses of function are described. *Arabidopsis* includes 15 WOX family members [55], seven of which have been subjected to genetic analyses (WUS1 [56]; PRESSED FLOWER/WOX3 [57]; WOX2 [55]; PRETTY FEW SEEDS2/WOX6 [58]; STIMPY/WOX9 [59]; WOX5 [60]; WOX4, J. Ji and M. J. Scanlon, unpublished data). Although the phenotypes of individual WOX mutants are varied (affecting shoot and root meristems, embryogenesis, and organogenesis of lateral organs and the vascular procambium), the combined genetic and molecular expression data suggest that an evolutionarily conserved general function of WOX proteins is to promote the organization of embryonic/meristematic cells or lateral organ initials. For example, WUS1 organizes proliferation in the central zone of *Arabidopsis* shoot meristems via repressing the transcription of several two-component response regulator genes *(ARR5, ARR6, ARR7,* and *ARR15),* which function to reduce SAM size [26].

Meanwhile, our analyses demonstrate that NS is required to repress the expression of two maize genes predicted to function in two-component signaling pathways that operate within the maize SAM (Figures 4A and 5E–5H; Table 1). These include a SAM-enriched response regulator-like gene (BG840771) as well as the maize histidine phosphotransfer gene *Zmhp1*, whose nonmutant RNA accumulation pattern (Figure 5E and 5G) mirrors that of the *ns* duplicate genes (Figure 1C). Taken together, these data suggest that transcriptional repression of specific, two-component signaling pathways may be conserved functions of WUS1 and NS. As likewise observed in our studies of ns meristems (Figure 5M–5P; Table 1), microarray analyses of WUS-induced gene expression also revealed the up-regulation of numerous jasmonate-induced *lectin* genes [26], including an apparent homolog of the maize jacalin-related *lectin* gene (DN210415), which is down-regulated in the ns mutant SAM. Although preliminary, these analyses suggest that NS and WUS1 may share a conserved WOX function to activate the expression of specific *lectin* genes during plant development. The LM-microarray analyses described herein provide a starting point toward reverse genetic and biochemical analyses of the mechanisms of NS-mediated founder cell recruitment during maize leaf initiation.

## Materials and Methods

### Plant materials.

The ns 1:1 line was propagated by crossing ns mutant plants (genotype *ns1-R* and *ns2-R*) onto nonmutant siblings (genotype *Ns1/ns1-R* and *ns2-R*) for over twenty successive generations as described [5,6]. Seedlings of this near-isogenic ns 1:1 line were grown in an environmentally controlled chamber with light intensity 220–250 μES^−1^M^−2^; 25 °C for 15 h of light; 20 °C for 9 h of dark; 50% for humidity, and harvested for LM at 2 wk after germination.

### LM of maize SAMs.

Seedlings were fixed in acetone and paraffin-embedded as described [16]. SAM cells were laser microdissected from 10-μm sections (ten to 12 sections per SAM) using the P.A.L.M. Laser Microbeam (http://www.palm-microlaser.com). Expression of the lateral SAM domain control gene *ns2* was highly enriched in our laser-dissected SAM samples compared to that of the whole seedlings, as monitored by RT-PCR and microarray (unpublished data). We used six biological replicates in these experiments, each comprised of ten to 12 whole ns or nonmutant laser microdissected SAMs (ranging from 2.4 mm^2^ to 4.2 mm^2^ of tissue) (Table S1). RNA was isolated using the PicoPure RNA extraction kit (Arcturus Molecular Devices, http://www.moleculardevices.com), and two rounds of RNA amplification were performed using T7-RNA polymerase as described [11] with changes described in [61]. Yields of amplified SAM RNA ranged from 16.7 μg to 57.6 μg quantities.

We reverse transcribed two μg of amplified SAM RNA with Superscript II (Invitrogen, http://www.invitrogen.com) and 0.5 μg of random primers (Roche Diagnostics, http://www.roche.com). The resultant cDNA were indirectly labeled with Cy dyes assisted by amino allyl incorporation as described [11]; dye bias was removed by swapping Cy dyes between the RNA samples. Microarray hybridizations were performed as described [11].

### SAM microarrays.

Two technical problems prevented our use of laser microdissected, amplified RNA in hybridizations with the maize oligo arrays that are currently in production at the University of Arizona (http://www.maizearray.org). First, amplified RNA prepared as described above is antisense orientated and therefore unusable with sense-directed oligo arrays. Although, linear RNA amplification protocols that generate sense-oriented RNA are available, these truncate the 3′ ends of the amplified RNA product. Because the majority of the maize oligo array sequences are generated from 3′ ends of maize ESTs, sense-amplified RNA generated by these protocols is also suboptimal. A second caveat is that the maize EST sequences that were used to design the maize oligo microarrays are underrepresented for sequences derived from the vegetative maize SAM; SAM-specific cDNA libraries were not deeply sequenced in previous maize EST projects. Therefore, we initiated a SAM EST discovery project in which cDNA was prepared from hand-dissected maize apices (SAM plus P1–P4) and 31,036 apex ESTs were generated [16] and submitted to GenBank (http://www.ncbi.nlm.nih.gov/Genbank). These include 3,503 SAM ESTs that are not found among EST libraries from any other maize tissues [16]; these SAM EST sequences are also not represented on the current maize oligo arrays.

In light of the obstacles preventing our use of the maize oligo arrays, three microarrays (SAM 1.1, SAM 2.0, and SAM 3.0) containing a combined total of 37,662 informative cDNAs including approximately 21,721 maize genes were constructed. SAM 1.1 contains 14,401 informative spots corresponding to ~9,423 maize cDNAs; SAM 2.0 contains 8,991 informative spots representing ~7,599 maize genes. Whereas SAM 1.1 and SAM 2.0 contain maize UniGenes [62] as well as cDNAs derived from maize inflorescences, the SAM 3.0 chip is particularly enriched for cDNAs derived from the maize SAM. Gene chip SAM 3.0 contains 14,270 informative spots and approximately 12,257 new genes, including more than 10,800 cDNAs derived from dissected maize apices and identified during this project (described above and in [16]). All three SAM microarrays contain 45 control genes that are known to be expressed in the maize SAM and/or young leaf primordia. SAM chips may be ordered online (http://www.plantgenomics.iastate.edu/maizechip), and their gene content may be searched via the online tool MADI (MicroArray Data Interface, http://schnablelab.plantgenomics.iastate.edu:8080/madi).

### Microarray analyses.

Hybridized arrays were scanned using a ScanArray 5000 (GSI Lumonics, http://www.gsilumonics.com) at 10-μm resolution. Image processing utilized Digital Genome System software (MolecularWare, http://www.calbatech.com). Signals were background corrected and LOWESS normalized within each slide to remove intensity-dependent dye bias [63]. Normalization across slides was accomplished by median centering data from each channel [64]. Our model for the normalized log-scale signal intensities for any given gene is as follows:

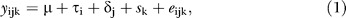
where y_ijk_ denotes the normalized log-scale signal intensity from SAM type i (i = 1,2 for mutant and nonmutant SAMs, respectively), dye j (j = 3,5 for Cy3 and Cy5 dyes, respectively), and slide k (k = 1,2,3,4,5,6); μ is an intercept term; τ_i_ denotes the effect of SAM type i; δ_j_ denotes the effect of dye j; *s*_k_ denotes the random effect of slide k; and *e*_ijk_ denotes a random residual term. The slide and residual random effects are assumed to be independent and normally distributed with a single variance for slides and a single variance for residuals. All parameters are allowed to vary from gene to gene though we have suppressed a gene-specific subscript to simplify notation.

To obtain tests of SAM type effects (*H*_0_ : τ_1_ = τ_2_), the difference between normalized signals (Cy5 minus Cy3) was computed for each spot. Based on our model (1) above, the six differences (denoted *d*_1_,...,*d*_6_) can be modeled as *d*_k_ = β_0_ + β_1_ *x*_k_ + ɛ_k_, where β_0_ = δ_5_ − δ_3_, β_1_ = τ_1_ − τ_2_, ɛ_k_ is a difference of the form *e*_ijk_ − *e*_i'j'k_, and *x*_1_ , ..., *x*_6_ are −1, −1, −1,1,1, and 1 to correspond to our design in which three of the slides have Cy3 and Cy5 assigned to mutant and nonmutant SAMs, respectively, and three have the opposite assignment. In this simple linear regression model, the intercept term accounts for gene-specific dye effects not removed in normalization (δ_5_ − δ_3_), and the slope term accounts for the SAM type effect of interest (τ_1_ − τ_2_), which corresponds to the mean difference in normalized log-scale expression between mutant and nonmutant SAMs. The resulting *p*-values from the tests for SAM type effects were converted to *q-*values using the method of Storey and Tibshirani [65] to estimate the false discovery rate (FDR) associated with any *p-*value threshold for significance. Functional annotation of differentially expressed genes was performed as described [18]. MIAME guidelines utilized in these experiments are described in Text S1; all microarray data are available at Gene Expression Omnibus (GEO; http://www.ncbi.nlm.nih.gov/geo).

### qRT-PCR and in situ hybridization.

qRT-PCR analyses were performed on cDNA synthesized from the identical SAM-amplified RNA samples used in our analyses, using either Taq-Man or SYBR-Green probes (Table S2) [66]. We used three biological replicates, upon which three technical replicates were performed. Reactions were normalized to control *ubiquitin* expression as described [67].

Tissue-specific qRT-PCR analyses were performed as above using the SYBR-Green methodology and gene-specific probes (Table S1); three technical replicates were performed. Expression of each transcript was normalized to the level of ubiquitin controls; relative gene expression values were graphed using the iQ5 Optical System Software version 1.0 (Bio-Rad, http://www.bio-rad.com), wherein no expression was assigned a value of zero. All tissues were derived from the maize inbred B73. Tissues included the laser microdissected SAM from 14-d-old seedlings; fully expanded mature leaf (leaf 10); fully expanded leaf from 14-d-old seedlings (leaf 4); seedling roots; immature ears (6-mm long) containing multiple spikelet meristems and spikelet pair meristems with glume primordia; and immature tassels (8 mm) containing branch primordia, multiple spikelet meristems, and spikelet pair meristems with glume primordia. Except for LM-derived SAM tissues (RNA extracted and amplified as above), all RNA extractions of maize tissues were performed using the Trizol method, as described [2].

Maize 14-d-old seedlings were grown in controlled conditions (above) and processed for in situ hybridization as described [49]. For each gene-specific probe analyzed, at least six replicate samples each of ns1-R mutant and nonmutant sibling were analyzed. Cartoons of transcript accumulation patterns modeled in Figures 5 and 6 depict SAM expression only. Expression in leaf primordia, which was not measured in our microarray analyses, is not depicted in cartoons.

## Supporting Information

Figure S1 qRT-PCR Analyses of Transcript Accumulation for Maize Genes CB816294 and DV490133 in Multiple Maize Tissues (92 KB PPT)

Figure S2 qRT-PCR Analyses of Transcript Accumulation for Maize Genes BI361046 and CB816286 in Multiple Maize Tissues (119 KB PPT)

Figure S3 qRT-PCR Analyses of Transcript Accumulation for Maize Genes DY576324 and DY400928 in Multiple Maize Tissues (116 KB PPT)

Figure S4 qRT-PCR Analyses of Transcript Accumulation for Maize Genes BG518167 and DN210415 in Multiple Maize Tissues (190 KB PPT)

Figure S5 qRT-PCR Analyses of Transcript Accumulation for Maize Genes CB381550 and DY402633 in Multiple Maize Tissues (80 KB PPT)

Table S1 LM Microarray Samples Used in This Study (37 KB DOC)

Table S2 Primers Used for qRT-PCR (41 KB DOC)

Text S1 MIAME Guidelines Checklist (11.5 MB XLS)

### Accession Numbers

The Gene Expession Omnibus (GEO) database (http://www.ncbi.nlm.nih.gov/geo) accession numbers for the genes discussed in this paper are *ns,* GSE7248; *knotted1,* AY260164; and *Zmhp1,* DN233962.

## Abbreviations

ARF: ADP-ribosylation factor
ARR: a RESPONSE REGULATOR
EST: expressed sequence tag
LM: laser microdissection
*ns,*: *narrow sheath;*
PIN: PINFORMED1 family of efflux proteins
qRT-PCR;: quantitative real-time-PCR
SAM: shoot apical meristem
WOX: WUSCHEL1-like homeobox
ZmRR: maize response regulator
