# Supplementary material for: Laser Microdissection of Narrow Sheath Mutant Maize Uncovers Novel Gene Expression in the Shoot Apical Meristem
Source: PLoS Genet. 2007 Jun 15;3(6):e101. doi: 10.1371/journal.pgen.0030101 (PMC1904365; doi:10.1371/journal.pgen.0030101)
Supplement: Supplementary file 1 [file pgen.0030101.st001.doc]

| **Supplemental table 1. Laser microdissection microarray samples used in this study** | | | | | | | | | | | | |
| --- | --- | --- | --- | --- | --- | --- | --- | --- | --- | --- | --- | --- |
| **Sample Information** | **ns1** | **wt1** | **ns2** | **wt2** | **ns3** | **wt3** | **ns4** | **wt4** | **ns5** | **wt5** | **ns6** | **wt6** |
| mm^2^ captured^i^ | 4.1 | 4.2 | 3.3 | 3.2 | 4.2 | 3.3 | 2.8 | 2.4 | 2.5 | 2.8 | 2.2 | 2.4 |
| aRNA yield (µg) | 47.3 | 41.2 | 31.4 | 28.9 | 16.7 | 31.6 | 55.3 | 21.6 | 57.6 | 22.1 | 30.8 | 17.1 |
| Labeling for SAM1 | Cy3 | Cy5 | Cy5 | Cy3 | Cy3 | Cy5 | Cy5 | Cy3 | Cy3 | Cy5 | Cy5 | Cy3 |
| Labeling for SAM2 | Cy5 | Cy3 | Cy3 | Cy5 | Cy5 | Cy3 | Cy3 | Cy5 | Cy5 | Cy3 | Cy3 | Cy5 |
| Labeling for SAM3 | Cy3 | Cy5 | Cy5 | Cy3 | Cy3 | Cy5 | Cy5 | Cy3 | Cy5 | Cy3 | Cy3 | Cy5 |

^i^ square surface area of SAM tissue isolated by laser microdissection
