# Supplementary material for: Laser Microdissection of Narrow Sheath Mutant Maize Uncovers Novel Gene Expression in the Shoot Apical Meristem
Source: PLoS Genet. 2007 Jun 15;3(6):e101. doi: 10.1371/journal.pgen.0030101 (PMC1904365; doi:10.1371/journal.pgen.0030101)
Supplement: Supplementary file 2 [file pgen.0030101.st002.doc]

| **Supplemental table 2. Primers used for quantitative RT-PCR** | | |
| --- | --- | --- |
| **Accession No** | **Forward primer 5'-3'** | **Reverse primer 5'-3'** |
| DY576324 | ATTGGGGAAGGTGCAGAAG | CACTTCAGTGACGGCTTCAA |
| DV491384 | TCTCCTCCAATCCAGCTCTC | TGCACTGCAAGTAGGGATTG |
| DY400928 | CGTACTTATTCAAGCCCTTCAGCAAGTA[FAM]G | AACTGAACTCCATGCTGAACTGAA |
| BG462729 | ACTCTGCCTCTGTGCTGGAA | ATTCTTGGCACTGGCAAAGC |
| CB816286 | CCCTGTCGGCGAGATACCTTC | CGTAGACTGACGAGTGGGCGATCTA[FAM]G |
| CD650947 | GAGCTGATGGTACTCGAAATGG | CGCTCAGACATCTTCAAGCAGATTGAG[FAM]G |
| BG518167 | AACACGATTTCGGTAAGCAGTT | CGGCAGGCTTCTGTTAGTTATT |
| BM380298 | CGTGTTCAAACCAATAGTCGAG | ACGCTCAGCTATCTGCCTATCT |
| CB816294 | ACCGGTGTCATGAAGAGAAGAA | GATCTTGGTGACGGAGGAGAT |
| DN205423 | CAGCGCTGTCAGTATAGGTGAA | CTCACAGTCAGAGCTGCAAGAT |
| DV942355 | GCCTAATGTGGGGAAAGGTT | GCCAATCGCTCTTTTGACTT |
| BI359303 | ATCCTACAGAGCGTGATTCCAT | TTTAGCGGGATTATTCGTGACT |
| BG946630 | ATCCTACAGAGCGTGATTCCAT | CTCTCTGGTCTGGAGGAGGTC |
| BI361046 | AAAATTCTCCCGAAAATCCAAT | GAGAAAATCGATTCATCAGACG |
| BI096810 | CATCTATGTCAATTAAAGGGACTAAAA | AGAGTATCGGCAAGGAATCTCG |
| BG840771 | TTTTTCTTAACTGGCCTGCATT | CATACGCTTAGGTGTTTGTGGA |
| DV490133 | CACAGATCATAGCCTTCAAATGAATCTG[FAM]G | TGCAGGGAACAGAGAAATAGCA |
| DV621960 | TTTTTCAAAATCCCATACTCATCC | TGACGATCGTTCGCATCC |
| DY402633 | GAGCCGCTGAACTTTACAGG | GAGTAGCGCCCTTTGTGAAC |
| DN210415 | GCTGTCAGTATAGGTGAACTC | GGACAAGAGATACGTGAGTG |
| CB381550 | TGCTACAGGAAAGTCAAGAG | CATTCACCTCAAACAAAGTCAG |
| AY260164 | GGCACTGGCTGAGTCTAC | CTCCTCGGATGGCTTCCAGT |
